# General practitioners’ clinical decision-making in patients that could have cancer: a vignette study comparing the Baltic states with four Nordic countries

**DOI:** 10.1080/02813432.2025.2451653

**Published:** 2025-01-21

**Authors:** Alexander Rosendahl, Anet Vanaveski, Liina Pilv-Toom, Jānis Blumfelds, Vija Siliņa, Mette Brekke, Tuomas Koskela, Aurimas Rapalavičius, Hans Thulesius, Peter Vedsted, Michael Harris

**Affiliations:** a Institute of Family Medicine and Public Health, University of Tartu, Tartu, Estonia; b Family Medicine Residency, University of Tartu, Tartu, Estonia; c Department of Family Medicine, Riga Stradiņš University, Riga, Latvia; d Department of General Practice, Institute of Health and Society, University of Oslo, Oslo, Norway; e Faculty of Medicine and Health Technology, Tampere University, Tampere, Finland; f Wellbeing Services County of Pirkanmaa, Tampere, Finland; g Department of Family Medicine, Lithuanian University of Health Sciences, Kaunas, Lithuania; h Department of Medicine and Optometry, Faculty of Health and Life Sciences, Linnaeus University, Växjö, Sweden; i Research Unit for General Practice, Aarhus, Denmark; j Department of Clinical Medicine, University Clinic for Innovative Patient Pathways, Aarhus University, Aarhus, Denmark; k Department for Health, University of Bath, Bath, UK; l Institute of Primary Health Care Bern (BIHAM), University of Bern, Bern, Switzerland

**Keywords:** Cancer, general practice, primary health care, early diagnosis, geography

## Abstract

**Objective:**

Relative one-year cancer survival rates in the Baltic states are lower than the European mean; in the Nordic countries they are higher than the mean. This study investigated the likelihood of General Practitioners (GPs) investigating or referring patients with a low but significant risk of cancer in these two regions, and how this was affected by GP demographics.

**Design:**

A survey of GPs using clinical vignettes.

**Setting:**

General Practice in Denmark, Estonia, Finland, Latvia, Lithuania, Norway, and Sweden.

**Subjects:**

General Practitioners.

**Outcome measures:**

A regional comparison of GPs’ stated immediate diagnostic actions (whether or not they would perform a key diagnostic test and/or refer to a specialist) for patients with a low but significant risk of cancer (between 1.2 and 3.6%).

**Results:**

Of the 427 GPs that completed the questionnaire, those in the Baltic states, and GPs that were more experienced, were more likely to arrange a key diagnostic test and/or refer their patient to a specialist than those in Nordic Countries or who were less experienced (*p* < 0.001 for both measures). Neither GP sex nor practice location within a country showed a significant association with these measures.

**Conclusion:**

While relative one-year cancer survival rates are lower in the Baltic states than in four Nordic countries, we found no evidence that this is due to their GPs’ reluctance to take immediate diagnostic action, as GPs in the Baltic states were more likely to investigate and/or refer at the first consultation. Research on patient and secondary care factors is needed to explain the survival differences.

## Introduction

In Europe, there is a large between-country variation in cancer survival rates, with the 1-year relative survival rate for all cancer sites varying from 58.2 to 81.1% [1]. Although the relative one-year cancer survival rate may be affected by lead-time bias (when a diagnostic approach identifies the disease earlier, but does not lead to a true improvement in survival), overdiagnosis and overtreatment (finding or treating screening-detected cancer that will never cause any symptoms) and differences in national cancer registration methods [2,3], it may also be due to longer diagnostic intervals and later stage at diagnosis and treatment start [4,5]. Overall, lower relative one-year survival rates are believed to be an indicator of more advanced disease at diagnosis [6,7]: more advanced cancers often have poorer outcomes [8], and for many cancers, the stage of disease at diagnosis is related to survival [9]. This means that earlier diagnosis is instrumental in helping to improve cancer survival [10].

However, it is unclear where and how this can be achieved [11], as late diagnosis may be due to patients presenting later (longer patient interval), late referrals from primary care (longer primary care interval), or waiting longer for appropriate diagnostics and treatment (longer system interval) [8]. This can be a particular problem when patients with cancer present without ‘red flag’ symptoms, and how General Practitioners (GPs) act is affected by how their health services are organised [12]. To be able to improve earlier cancer detection rates, we need to examine which aspects of patient management affect earlier diagnosis in a primary care setting. This is particularly relevant to GPs, as the most frequently missed diagnoses causing harm in primary care are cancers [13].

The aim of this exploratory vignette-based study was to investigate whether the management of patients with a low but significant risk of cancer in a primary care setting differed between the Baltic states and four Nordic countries. The Baltic and Nordic regions, although close geographically, have developed differently and their nations have different primary care systems, so we hypothesised that the poorer cancer survival rates in the Baltic states may be due to a lower likelihood of immediate diagnostic action by their GPs. The study also examined how the management of these patients was affected by GP demographic factors, as these have been found to affect GPs’ overall referral rates [14–16].

## Material and methods

### Design

The Örenäs Research Group (ÖRG), a European group of primary care cancer researchers [17], performed a 23-country European online cross-sectional survey of GPs to identify the factors associated with national variations in cancer survival [18]. In the present study, we use the data from the northern European countries that participated in the study: Estonia, Latvia, Lithuania (the Baltic states), and Denmark, Finland, Norway, and Sweden (four Nordic countries). A sample of GPs in these countries completed an online questionnaire with clinical vignettes that described patients presenting with specific symptoms that may be due to cancer and were asked what action they would take.

### Outcome measures

The primary outcome was a between-region comparison of GPs’ stated immediate actions, in terms of whether or not they would perform a key diagnostic test and/or refer to a specialist.

The secondary outcome was the relationship between GPs’ demographics (years since graduation, sex, and location of practice) and their immediate diagnostic action rates.

### Study population

General Practitioners.

### Development of the questionnaire

We used clinical vignettes as they have been validated as a measure of clinical practice [19,20]. Vignette methods have been shown to accurately measure practice variation [21], and are a valuable way to assess practices in health care systems to make direct comparisons and identify disparities in those practices [22]. Following a literature review, ÖRG investigators developed a questionnaire designed to elicit GPs’ demographic information and their actions for patients who could have cancer. The questionnaire with five clinical vignettes (three new vignettes and two previously designed and validated by the International Cancer Benchmarking Partnership (ICBP) [23] and used with their permission) was piloted by the ÖRG to assess validity. More information about this process is given elsewhere [24]. One of the vignettes was found to be invalid and was removed. The next version was then piloted by 49 GPs in 16 ÖRG member countries. No changes to the questions were made following this second pilot.

National ÖRG leads arranged for translations of the questionnaire from English into local languages. Translation and validation by back-translation were done in a standardised way [25] and are described elsewhere [26].

### Description of the questionnaire

The questionnaire consisted of 47 items divided into four sections: (a) demographic questions (years since graduation, gender, urban/rural/island/mixed location of practice), (b) referral availability questions (tests and specialist opinions either directly or indirectly available to the respondent), (c) four fictitious clinical vignettes, and (d) 20 health system factor questions. The vignettes provided information on the patient’s presenting symptoms, previous medical history, medication, clinical findings, and other relevant information. A factor analysis of the results of the survey section on the effect of health system factors has been reported separately [27].

The questionnaire for GPs in England, on which those for the other countries were based, is given in Supplementary Appendix 1. The survey was answered anonymously to decrease the social desirability of responses to questions that may be considered sensitive [28].

The four clinical vignettes described consultations with patients having a low but significant possibility (between 1.2 and 3.6%) of a specific type of cancer: lung, ovarian, breast, or colon cancer (summarised in Table 1).

**Table 1.** Summary of the clinical vignettes.

A 62-year-old male smoker with chronic obstructive pulmonary disease and a 2-week history of a productive cough; positive predictive value (PPV) for lung cancer: 3.6% [29]. 53-year-old woman with lower abdominal pain and abdominal distension; PPV for ovarian cancer: 3.1% [30]. A 35-year-old breastfeeding woman with an abnormal nipple discharge and eczematous changes around the nipple; PPV for breast cancer: 1.2% [31]. A 22-year-old man with coeliac disease who now has abdominal pain, rectal bleeding, and diarrhoea; PPV for colorectal cancer: 3.4% [29].

For each patient, five possible immediate management decisions were given, with an invitation to choose as many as needed:

‘Which of these would you do AT THIS CONSULTATION?’

- ‘I would write an appropriate prescription for the patient’.
- ‘I would arrange to see the patient again for follow-up and reassessment’.
- ‘I would not arrange formal follow-up, but would tell the patient under what circumstances they should see me again’.
- ‘I would organise an investigation [a test] at this consultation’.
- ‘I would refer the patient to a specialist at this consultation’.

Those who chose to investigate the patient were given a range of possible diagnostic tests to choose from. The primary interest was to evaluate a GPs’ management choice that would be likely to identify a cancer as a cause of the patients’ symptoms, by either requesting a relevant diagnostic test or by referring to a specialist. These criteria, including the relevance of the diagnostic tests, were decided by consensus in the researcher team, all of whom were GPs.

### Sample size and recruitment of participants

The national ÖRG leads were asked to recruit at least 50 participants, with no maximum limit, allowing recruitment of a varied sample with regard to gender, years since graduation, and site of practice. The possibility of cancer as being a cause of the vignette symptoms was not mentioned in either the recruitment email or the survey, which stated that the aim was to ‘identify which factors affect GPs’ decisions to refer patients for further investigation’.

Consent was implied by agreeing to take part in the survey. All data were collected anonymously.

The questionnaire was designed using SurveyMonkey (SurveyMonkey, California, USA). Because of the study’s wide geographical coverage, we used online delivery; this has previously been successfully used in research involving cancer care professionals [32,33]. National leads were asked to send two follow-up reminders to encourage completion of the survey.

### Statistical analysis

We use descriptive statistics to report demographic data, practice characteristics, and GPs’ actions.

For each individual GP, we aggregated the decision-making rates from the four individual vignette responses: if a GP referred all four cases, we assigned a referral rate of 1. If a GP referred 3 out of the 4, we assigned a referral rate of 0.75; if 2 out of the 4, a rate of 0.5; if 1 out of the 4, a rate of 0.25; and if none out of 4, a rate of zero. The outcome was therefore an ordinal variable. Merging the vignette data has face validity as the aim was to explore GPs’ diagnostic actions in patients with symptoms that could be indicative of cancer, and the four cancer diagnoses account for 37% of new cancer cases in Europe [34]. Other investigators have also merged vignette data where the aim is to compare the action of different groups of healthcare professionals, as in this study, as opposed to comparing the effect of different vignettes on their action [35,36].

As it was considered that some GPs would not organise a diagnostic test because they were referring to a specialist, and conversely some GPs would not refer to a specialist because they were organising a diagnostic test, we used a composite measure of a decision to arrange a diagnostic test and/or refer to a specialist, that is, the likelihood of taking immediate diagnostic action for cancer.

Because the outcome was an ordinal variable, we used multiple regression to estimate the associations between GPs’ referral and/or diagnostic actions and: geographical region, GP sex, years of experience, and practice location. The multiple regression method considers the effect of each of these exploratory factors on our outcome of interest (GPs’ referral and/or diagnostic actions), and it evaluates the relative effect of each of these exploratory factors on the outcome when holding all the other factors in the model constant. Years of experience was based on years since graduation: <10 years = 1; 10–19 years = 2; 20–29 years = 3; 30–39 years = 4; ≥40 years = 5. ‘Sex’ was based on: Female = 1; Male = 2. Urban practices were most frequent and were chosen as the reference factor for practice location.

Tests were 2-tailed, with statistical significance defined as *p* ≤ 0.05. Calculations were performed using IBM SPSS v28.

## Results

In total, 427 GPs completed the questionnaire: 134 (31.4%) from the Baltic States (Estonia 64, Latvia 41, Lithuania 29), 293 from the Nordic countries (Denmark 90, Norway 81, Sweden 64, Finland 58). The response rates, where known, are given in Table 2.

**Table 2.** National response rates.

Country	Number who completed the questionnaire	Number invited	Response rate (%)
Estonia	64	Not known	Not known
Latvia	41	Not known	Not known
Lithuania	29	Not known	Not known
Denmark	90	400	22.5
Finland	58	178	32.6
Norway	81	500	16.2
Sweden	64	400	16.0

### Demographics

Just under half (201 participants, 47.1%) had graduated within the last 20 years (Table 3). A majority (277, 64.9%) were female, and almost two-thirds (275, 64.4%) of practices were in an urban location.

**Table 3.** Respondent demographics.

	Number of respondents (% of all respondents)
Total	427 (100)
Region	
Baltic	134 (31.4)
Nordic	293 (68.6)
Years since qualifying	
<10 years	97 (22.7)
10–19 years	104 (24.4)
20–29 years	91 (21.3)
30–39 years	117 (24.4)
≥40 years	17 (4.0)
Unknown	1 (0.2)
Sex	
Female	277 (64.9)
Male	149 (34.9)
Unknown	1 (0.2)
Practice location	
Urban	275 (64.4)
Rural	84 (19.7)
Island/remote	6 (1.4)
Mixed	61 (14.3)
Unknown	1 (0.2)

### Likelihood of GP organising a key diagnostic test at this consultation

GPs in the Baltic states, and more experienced GPs, were more likely to investigate these patients at the first consultation (Table 4). Neither GP sex nor practice location within a country showed a significant association.

**Table 4.** Multiple regression for likelihood of GP investigating the patient.

	Unstandardised coefficients		Standardised coefficients	*t*	*p*-Value
	*B*	Std. Error	Beta		
(Constant)	0.711	0.037		19.403	<0.001
Region: Nordic countries	−0.267	0.030	−0.423	−9.307	<0.001
Years since graduation	0.049	0.010	0.201	4.689	<0.001
Sex: male	−0.036	0.028	−0.058	−1.268	0.205
Location: rural	0.011	0.032	0.016	0.357	0.721
Location: island/remote	−0.154	0.107	−0.062	−1.439	0.151
Location: mixed	0.040	0.037	0.047	1.060	0.290

*Note:* In Tables 4–6, the Beta values indicate how much each exploratory factor makes to explaining the dependent variable (in this table, likelihood of GP investigating the patient) when the variance explained by all other exploratory factors in the model is controlled for. A *p*-value of <0.05 indicates that the exploratory factor makes a statistically significant unique contribution to the model.

In Tables 4–6, urban practices are the reference factor for practice location.

**Table 5.** Multiple regression for likelihood of GP referring the patient to a specialist.

	Unstandardized coefficients		Standardised coefficients	*t*	*p*-Value
	*B*	Std. Error	Beta		
(Constant)	0.912	0.040		20.552	<0.001
Region: Nordic countries	−0.313	0.032	−0.469	−9,842	<0.001
Years since graduation	−0.004	0.011	−0.015	−0.347	0.729
Sex: male	0.006	0.030	0.009	0.191	0.849
Location: rural	−0.030	0.035	−0.038	−0.857	0.392
Location: island/remote	0.106	0.115	0.040	0.923	0.357
Location: mixed	0.055	0.040	−0.062	1.355	0.176

**Table 6.** Multiple regression for likelihood of GP taking immediate diagnostic action (arranging a key diagnostic test and/or referring to a specialist).

	Unstandardised coefficients		Standardised coefficients	*t*	*p*-Value
	*B*	Std. Error	Beta		
(Constant)	0.853	0.028		30.570	<0.001
Region: Nordic countries	−0.196	0.022	−0.418	−8.721	<0.001
Years since graduation	0.024	0.008	0.133	3.024	0.003
Sex: male	−0.010	0.021	−0.023	−0.481	0.631
Location: rural	0.026	0.025	0.047	1.046	0.296
Location: island/remote	−0.044	0.081	−0.024	−0.544	0.587
Location: mixed	0.020	0.028	0.032	0.699	0.485

### Likelihood of GP referring the patient to a specialist

GPs in the Baltic states were more likely to refer these patients to a specialist immediately (Table 5). Other demographic factors showed no significant association.

### Likelihood of GP taking immediate diagnostic action (arranging a key diagnostic test and/or referring to a specialist)

GPs from the Baltic states were significantly more likely to take immediate diagnostic action, as were more experienced GPs (Table 6). Neither GP sex nor practice location showed a significant association.

## Discussion

### Principal findings

In vignettes representing patients with a low but significant risk of cancer in a primary care setting, GPs in the Baltic states stated they were more likely to take immediate diagnostic action (referring and/or arranging a key diagnostic test) than their colleagues in Nordic countries.

More experienced GPs (in terms of years since graduation) were more likely to take diagnostic action at the initial consultation. GP sex and practice location showed no significant association with the likelihood of diagnostic action.

### Interpretation of the results

These findings show no evidence that the lower relative one-year cancer survival rates in the Baltic states are associated with diagnostic delays on the part of their GPs. It may be that, in the Baltic states, patients present later and have generally poorer health, resulting in their GPs being more used to making rapid investigation and referral decisions. It is also possible that GPs in the Baltic states refer patients to specialists earlier than in the Nordic countries because a potential diagnosis of cancer is higher in their list of differential diagnoses for other reasons, or it may be that they are less able to directly refer patients for computed tomography (CT) and magnetic resonance imaging (MRI) investigations, so they refer patients who may need these.

Our finding that GPs who had more years since graduation were more likely to take diagnostic action could be because of differences in education in the intervening years, or it may be that their additional years of clinical experience heightened their suspicions as to sinister diagnoses or increased their diagnostic decisiveness.

### Strengths and limitations of the study

This study surveyed GPs who work in areas that differ in their geography, socioeconomic factors, and healthcare systems. The questionnaire was carefully developed and piloted by GPs, with vignettes that have previously been shown to be effective in multi-national studies. The translated questionnaires were validated by GPs in all participating countries. Respondent anonymity may have reduced the risk of social desirability bias.

A strength of the study is its vignette methodology: it is a valuable way to compare the quality of care across several countries [33]. However, the vignettes designed by the researchers may not reflect GPs’ experiences of actual patients with the condition [37]: the vignettes may have underestimated the variability associated with the medical conditions, and to ensure that respondents were not overloaded with information, they focussed on selected symptoms without providing a broader context of patients’ medical histories. While respondents’ answers may have been biased, as they may have stated actions that they would not have taken in real life, we are unable to postulate how this would have affected the findings, and vignettes have been shown to outperform both chart abstraction (medical record review) [20,38] and direct observation [39].

The recruitment methods used in this study varied by country, resulting in different response rates. While low survey response rates are common in primary care [40] and are known to vary between countries, the response rates in our study were comparable to those of a recent ICBP survey, in which response rates varied from 5.5 to 45.6% [41]. The respondents may not have been representative of their regions’ GPs. We have no data on non-responders because the survey was anonymous, and it may be that GPs with a greater interest in this subject were more likely to complete the questionnaire. There may have been national differences in response styles, for instance, choosing or avoiding the ‘extreme’ options on the scale [42], but as the translation process also included any necessary cultural adaptation, we believe that this bias was minimised.

### Comparison with existing literature

Studies like ours have not previously been performed in the Baltic States, so we are unable to make direct comparisons. A recent study in the Balkan region investigated GPs’ clinical decision-making in patients who could have lung cancer, and it found no association between GP demographics or practice characteristics with the decision to investigate or refer [43]. It did, however, find significant between-country differences regarding GPs’ level of direct access to investigations. Although our study showed that GPs with more years of clinical experience were more likely to take immediate diagnostic action, the opposite had been found in another study [14]; however, that study did not specifically consider cancer diagnosis as a variable.

In a study examining cancer stage and survival rates across European regions, a lower percentage of Eastern European patients were diagnosed at an early stage, while a higher percentage were diagnosed at a metastatic stage [44]. Even after adjusting for age, sex, and stage, Eastern European patients still had poorer survival, suggesting that late diagnosis alone does not account for the poorer cancer survival rates in Eastern Europe.

Our findings are in contrast to those of another international primary care vignette survey that showed a positive correlation between the readiness of GPs to investigate symptoms indicative of cancer and cancer survival rates [41]. However, that study included countries with comparable wealth, whereas our study investigated two regions with different levels of wealth and healthcare spending.

### Implications for research and practice

Having found no evidence that delayed referral from primary care is a reason for lower cancer survival in the Baltic States, there is a need to investigate other possible reasons, for example, patient or secondary care factors. These should include factors like levels of health education, uptake of screening programmes, and ease of access to primary care. Research is also needed to find out why GPs who are less experienced are less likely to take diagnostic action earlier in patients presenting with symptoms that could be indicative of cancer.

## Conclusions

This study used vignettes to investigate how GPs investigated or referred patients with a low but significant risk of cancer at their first consultations in the Baltic and Nordic regions, and how these actions were affected by GP demographics. While relative one-year cancer survival rates are lower in the Baltic states than in the Nordic countries, we found no evidence that this was associated with any delays in their GPs’ diagnostic actions. Indeed, GPs in the Baltic states were more likely to investigate and/or refer these vignette patients at the first consultation. GPs with more years of clinical experience were more likely to take immediate diagnostic action in these patients.

## Supplementary Material

Appendix 1 English language version of the questionnaire.pdf

Ethical and other approvals obtained.pdf

## Data Availability

To avoid the risk of identification of individual participants or patients, the datasets generated and analysed during the current study are not publicly available. However, they are available (with any identifying information redacted) from the corresponding author on reasonable request.
