# Supplementary material for: General practitioners’ clinical decision-making in patients that could have cancer: a vignette study comparing the Baltic states with four Nordic countries
Source: Scand J Prim Health Care. 2025 Jan 21;43(2):403–10. doi: 10.1080/02813432.2025.2451653 (PMC12090267; doi:10.1080/02813432.2025.2451653)
Supplement: Appendix 1 English language version of the questionnaire.pdf [file IPRI_A_2451653_SM7301.pdf]

The Örenäs Survey for Primary Care Doctors in England

Introduction

Thank you for taking part in this survey for primary care doctors.

The survey has been organised by the Örenäs Research Group, a Europe-wide group of family doctors who are interested in researching the factors that affect the way patients are managed in primary care.

The survey will take about 15 minutes to complete.

Your answers are confidential and we do not ask for your name or any contact details.

If you are happy to take the survey, please click on the "Next" link below.

Many thanks,
Michael Harris MB BS FRCGP, University of Bath

The Örenäs Survey for Primary Care Doctors in England

General information about you

1. How many years is it since you graduated as a doctor?

- ◯ Under 10 years
- ◯ 10-19 years
- ◯ 20-29 years
- ◯ 30-39 years
- ◯ 40 years or over
- ◯ I prefer not to say

2. Are you -

- ◯ Female?
- ◯ Male?
- ◯ I prefer not to say

3. What is your speciality? You can tick more than one if needed.

- ☐ Ear, nose & throat
- ☐ General practice/family medicine
- ☐ Internal/general medicine
- ☐ Obstetrics/gynaecology
- ☐ Oncology
- ☐ Orthopaedics
- ☐ Paediatrics
- ☐ Other (please specify)

4. What type of practice do you work in?

- ◯ Urban
- ◯ Rural
- ◯ Island
- ◯ Mixed

5. How many doctors work in your practice/health centre in total?

- ◯ 1
- ◯ 2
- ◯ 3
- ◯ 4-5
- ◯ 6-7
- ◯ 8-9
- ◯ 10 or more

Information about tests and specialist opinions that are available to you

6. Which of these tests are available to you…

	… in your own practice?	… at your request outside your practice	… not directly available to you, or only available via a specialist?
Microbiology tests (e.g. sputum and urine microscopy/culture)	☐	☐	☐
Abdominal diagnostic ultrasound	☐	☐	☐
Pelvic diagnostic ultrasound	☐	☐	☐
Gastroscopy	☐	☐	☐
Sigmoidoscopy	☐	☐	☐
Colonoscopy or CT colonography	☐	☐	☐
Plain X-ray (e.g. chest X-ray)	☐	☐	☐
Contrast X-ray (e.g. barium meal)	☐	☐	☐
MRI scan	☐	☐	☐
CT scan	☐	☐	☐
PET CT scan	☐	☐	☐
Mammography	☐	☐	☐

7. Which (if any) of these specialist opinions are available to you IN YOUR OWN PRACTICE? Tick as many as you like.

	I am qualified in this speciality	One or more colleagues in my practice is qualified in this speciality
Ear, nose & throat	☐	☐
General practice/family medicine	☐	☐
Internal/general medicine	☐	☐
Obstetrics/gynaecology	☐	☐
Oncology	☐	☐
Orthopaedics	☐	☐
Paediatrics	☐	☐

Other (please explain below)

The Örenäs Survey for Primary Care Doctors in England

First clinical case: Alexander

Alexander is 62 years old. He has had a respiratory tract infection for two weeks, with increased sputum production and increased use of his salbutamol inhaler. He has recently had a constant ache in his left shoulder.
He had chronic obstructive pulmonary disease (COPD) diagnosed by spirometry 2 years ago.
There is no other relevant past medical history.
He smokes 20 cigarettes a day and has done for over 40 years.
His current treatment is tiotropium, 1 dose daily, and a salbutamol inhaler for use as required.
There are no significant findings on physical examination and he is not acutely unwell.
NB: There are no right or wrong answers for this or any of the other scenarios, and this is not a test – we are interested in what doctors actually do in real life.
If any of the clinical cases aren't relevant to you because of your speciality, just click "Next" to go to the next page.

8. Which of these would you do AT THIS CONSULTATION?
Tick as many as needed.

- ☐ I would write an appropriate prescription for the patient.
- ☐ I would arrange to see the patient again for follow-up and reassessment.
- ☐ I would not arrange formal follow-up, but would tell the patient under what circumstances he should see me again.

9. Would you organise an investigation AT THIS CONSULTATION?

- ○ Yes
- ○ No

10. Would you refer the patient to a specialist AT THIS CONSULTATION?

- ○ Yes
- ○ No

Alexander: investigations

11. If Alexander came to see you today, which (if any) of these diagnostic actions would you be most likely to take AT THIS CONSULTATION?
You can tick as many or as few as you wish, or none at all.

- ☐ I would organise a base-line blood test, e.g. full blood count, creatinine.
- ☐ I would organise a specialist blood test (e.g. a tumour marker).
- ☐ I would organise a basic dipstick urine test.
- ☐ I would organise a microbiology test (e.g. sputum microscopy/culture).
- ☐ I would organise a plain X-ray.
- ☐ I would organise spirometry.
- ☐ I would organise one or more other special investigations or imaging (please specify below):

Second clinical case: Maria

A 53 year-old woman, whose last menstrual period was 6 months ago, has had colicky right lower abdominal pain for three weeks. She has noticed that her abdomen seems swollen and she has urinary frequency. She has had no change in the frequency or consistency of her stools (faeces).
She has had the same sexual partner for 20 years.
She is a frequent attender, often with complaints that remain undiagnosed.
There are no significant findings on abdominal examination and she is not acutely unwell.

12. Which of these would you do AT THIS CONSULTATION?
Tick as many as needed.

- ☐ I would write an appropriate prescription for the patient.
- ☐ I would arrange to see the patient again for follow-up and reassessment.
- ☐ I would not arrange formal follow-up, but would tell the patient under what circumstances she should see me again.

13. Would you organise an investigation AT THIS CONSULTATION?

- ◯ Yes
- ◯ No

14. Would you refer the patient to a specialist AT THIS CONSULTATION?

- ◯ Yes
- ◯ No

Maria: investigations

15. If Maria came to see you today, which (if any) of these diagnostic actions would you be most likely to take AT THIS CONSULTATION?
You can tick as many or as few as you wish, or none at all.

- [ ] I would organise a base-line blood test, e.g. full blood count, creatinine.
- [ ] I would organise a specialist blood test (e.g. a tumour marker).
- [ ] I would organise a basic dipstick urine test.
- [ ] I would organise a microbiology test (e.g. sputum microscopy/culture).
- [ ] I would organise a plain X-ray.
- [ ] I would organise a diagnostic ultrasound.
- [ ] I would organise one or more other special investigations or imaging (please specify below):

Second clinical case: Victoria

Victoria is a 35 year-old who is breast-feeding. She has pain in her left breast. It is not related to her menstrual cycle. She has recently noticed a small amount of abnormal discharge from her nipple, with some eczema around the nipple.
She has a long history of atopic dermatitis, which has only been on her elbows and knees and has been treated by emollients.
She has lost weight rapidly but she is still heavier than she was before her pregnancy.
Her mother had a mastectomy for breast cancer 5 years ago, but she was told that this was not familial.
Clinical examination confirms eczematous change around the nipple but there are no abnormalities palpable in the breast.

16. Which of these would you do AT THIS CONSULTATION?
Tick as many as needed.

- [ ] I would write an appropriate prescription for the patient.
- [ ] I would arrange to see the patient again for follow-up and reassessment.
- [ ] I would not arrange formal follow-up, but would tell the patient under what circumstances she should see me again.

17. Would you organise an investigation AT THIS CONSULTATION?

- ○ Yes
- ○ No

18. Would you refer the patient to a specialist AT THIS CONSULTATION?

- ○ Yes
- ○ No

Victoria: investigations

19. If Victoria came to see you today, which (if any) of these diagnostic actions would you be most likely to take AT THIS CONSULTATION?
You can tick as many or as few as you wish, or none at all.

- [ ] I would organise a base-line blood test, e.g. full blood count, creatinine.
- [ ] I would organise a specialist blood test (e.g. a tumour marker).
- [ ] I would organise a basic dipstick urine test.
- [ ] I would organise a microbiology test (e.g. skin swab for microscopy/culture).
- [ ] I would organise an ultrasound of the breast.
- [ ] I would organise a mammogram.
- [ ] I would organise one or more other special investigations or imaging (please specify below):

Fourth clinical case: Peter

Peter is 22 years old. Over the past 4 weeks, he has had increasing episodes of abdominal pain and rectal bleeding, often with diarrhoea. He has sometimes had rectal bleeding before but he has never been worried about it.
Two years ago he was diagnosed as having coeliac disease, and he has had a strict gluten-free diet since then.
His diet has not changed recently, but he has lost 5kg of weight.
There are no significant findings on physical examination and he is not acutely unwell.

20. Which of these would you do AT THIS CONSULTATION?
Tick as many as needed.

- ☐ I would write an appropriate prescription for the patient.
- ☐ I would arrange to see the patient again for follow-up and reassessment.
- ☐ I would not arrange formal follow-up, but would tell the patient under what circumstances he should see me again.

21. Would you organise an investigation AT THIS CONSULTATION?

- ○ Yes
- ○ No

22. Would you refer the patient to a specialist AT THIS CONSULTATION?

- ○ Yes
- ○ No

Peter: investigations

23. If Peter came to see you today, which (if any) of these diagnostic actions would you be most likely to take AT THIS CONSULTATION?
You can tick as many or as few as you wish, or none at all.

- [ ] I would organise a base-line blood test, e.g. full blood count, creatinine.
- [ ] I would organise a specialist blood test (e.g. a tumour marker).
- [ ] I would organise a basic dipstick urine test.
- [ ] I would organise a microbiology test (e.g. faeces microscopy/culture).
- [ ] I would organise a plain X-ray.
- [ ] I would organise a diagnostic ultrasound.
- [ ] I would organise a sigmoidoscopy.
- [ ] I would organise a colonoscopy or CT colonography.
- [ ] I would organise one or more other special investigations or imaging (please specify below):

On these and the following pages, we would like to know what affects your thinking with regard to referring patients like the ones on the previous pages for special investigations or to see a specialist.
Again, there are no right or wrong answers.

24. Guidelines and local systems

	Strongly agree	Agree	Neither agree nor disagree	Disagree	Strongly disagree
Common presentations are covered by local or national guidelines that usually give advice on which patients to refer.	○	○	○	○	○
The local health system encourages us to refer any patients with possible cancer early, even if there is a low risk of cancer.	○	○	○	○	○
In my practice, patients often have to travel a long way to see a specialist.	○	○	○	○	○

25. Relationship with specialist colleagues

	Strongly agree	Agree	Neither agree nor disagree	Disagree	Strongly disagree
I am able to refer directly to a named specialist.	○	○	○	○	○
I am able to refer to a specialist that I know personally.	○	○	○	○	○
I can easily telephone (or email) a specialist for informal discussion and advice.	○	○	○	○	○
Here, specialists usually welcome referrals.	○	○	○	○	○

26. Financial costs

	Strongly agree	Agree	Neither agree nor disagree	Disagree	Strongly disagree
Seeing a specialist can be a problem for some of my patients because of the financial cost to them.	○	○	○	○	○
We have a budget or quota (maximum limit) for diagnostic tests.	○	○	○	○	○
Here, high quality care for an individual patient is always more important than costs.	○	○	○	○	○
Referring or not referring doesn't affect me at all financially.	○	○	○	○	○
Referral costs are usually paid by insurance companies, not hospital or primary care budgets.	○	○	○	○	○

27. Concerns about complaints

	Strongly agree	Agree	Neither agree nor disagree	Disagree	Strongly disagree
My colleagues sometimes criticise me if I have referred a patient to them, but they think that I should have been able to manage the patient myself.	○	○	○	○	○
In general, patients prefer a GP, rather than a specialist, to look after them.	○	○	○	○	○

28. Waiting lists

	Strongly agree	Agree	Neither agree nor disagree	Disagree	Strongly disagree
We have access to a fast-track specialist appointment system for patients with suspected cancer.	○	○	○	○	○
Patients can self-refer to specialists, so GPs don't need to act as gatekeepers.	○	○	○	○	○

29. The effect of your workload

	Strongly agree	Agree	Neither agree nor disagree	Disagree	Strongly disagree
I am usually very busy, so I sometimes refer to help reduce my workload.	○	○	○	○	○
I usually have enough time in the consultation to think carefully about whether the patient needs a referral.	○	○	○	○	○

30. Patient and doctor feelings

	Strongly agree	Agree	Neither agree nor disagree	Disagree	Strongly disagree
I am likely to refer if the patient says that she/he would like to be referred, even if there are no “red flags”.	○	○	○	○	○
We are under media (newspaper, television) or public pressure to refer earlier.	○	○	○	○	○

Final page...

31. The last question - how do you think the speed of diagnosis of cancer in primary care could be improved?

Thank you for having completed this survey.
Michael Harris, for the Örenäs Research Group
