# Supplementary material for: General practitioners’ clinical decision-making in patients that could have cancer: a vignette study comparing the Baltic states with four Nordic countries
Source: Scand J Prim Health Care. 2025 Jan 21;43(2):403–10. doi: 10.1080/02813432.2025.2451653 (PMC12090267; doi:10.1080/02813432.2025.2451653)
Supplement: Ethical and other approvals obtained.pdf [file IPRI_A_2451653_SM7300.pdf]

Supplementary file. Ethical and other approvals obtained in each Örenäs Research Group participating jurisdiction

	Date of Ethics Approval	Approvals obtained	Reference
Estonia	9 December 2020	Research Ethics Committee of the University of Tartu.	322/T-20
Latvia	17 December 2020	Rigas Stradina universitates Petijumu ētikas komitejas.	Nr.6-1/13/2020/15
Lithuania	N/A	In Lithuania, research ethics approval was not required as the research did not involve any vulnerable subjects.	
Denmark	7 May 2016	Danish Data Protection Agency; according to Danish law and the Central Denmark Region Committees on Health Research Ethics, approval by the National Committee on Health Research Ethics was not required as no biomedical intervention was performed.	2009-41-3471
Finland	16 November 2016	Academic Ethics Committee of the Tampere Region.	16 November 2016
Norway	N/A	In Norway, research ethics approval was not required as no biomedical intervention was performed.	
Sweden	N/A	In Sweden, research ethics approval was not required as no biomedical intervention was performed. It does not fall under the law of research on human subjects to ask professionals about their work and how they perceive it.	
